# FGFR4 Profile as a Prognostic Marker in Squamous Cell Carcinoma of the Mouth and Oropharynx

**DOI:** 10.1371/journal.pone.0050747

**Published:** 2012-11-30

**Authors:** Roberta Lelis Dutra, Marcos Brasilino de Carvalho, Marcelo dos Santos, Ana Maria da Cunha Mercante, Diana Gazito, Rafael de Cicco, GENCAPO Group, Eloiza Helena Tajara, Iúri Drumond Louro, Adriana Madeira Álvares da Silva

**Affiliations:** 1 Faculdade de Medicina, Universidade de São Paulo,São Paulo, São Paulo, Brazil; 2 Laboratório de Biologia Molecular, Hospital Heliópolis, São Paulo, São Paulo, Brazil; 3 Serviço de Cirurgia Cabeça e Pescoço, Hospital Heliópolis, São Paulo, São Paulo, Brazil; 4 Programa de Pós Graduação em Biotecnologia, Universidade Federal do Espírito Santo, Vitória, Espírito Santo, Brazil; 5 Departamento de Anatomia Patológica, Hospital Heliópolis, São Paulo, São Paulo, Brazil; 6 Laboratório de Sequenciamento, Associação Beneficente de Coleta de Sangue, São Paulo, São Paulo, Brazil; 7 Instituto do Câncer, São Paulo, São Paulo, Brazil; 8 Head and Neck Genome Project, GENCAPO, Ribeirão Preto, São Paulo, Brazil; 9 Departamento de Biologia Molecular, Faculdade de Medicina de São José do Rio Preto, São José do Rio Preto, São Paulo, Brazil; 10 Departamento de Ciências Biológicas, Universidade Federal do Espírito Santo, Vitória, Espírito Santo, Brazil; 11 Departamento de Biologia, Universidade Federal do Espírito Santo, Alegre, Espírito Santo, Brazil; Barts & The London School of Medicine and Dentistry, Queen Mary University of London, United Kingdom

## Abstract

**Background:**

Fibroblast growth factor receptor 4 (FGFR4) is a member of a receptor tyrosine kinase family of enzymes involved in cell cycle control and proliferation. A common single nucleotide polymorphism (SNP) Gly388Arg variant has been associated with increased tumor cell motility and progression of breast cancer, head and neck cancer and soft tissue sarcomas. The present study evaluated the prognostic significance of FGFR4 in oral and oropharynx carcinomas, finding an association of FGFR4 expression and Gly388Arg genotype with tumor onset and prognosis.

**Patients and Methods:**

DNA from peripheral blood of 122 patients with oral and oropharyngeal squamous cell carcinomas was used to determine FGFR4 genotype by PCR-RFLP. Protein expression was assessed by immunohistochemistry (IHC) on paraffin-embedded tissue microarrays.

**Results:**

Presence of allele Arg388 was associated with lymphatic embolization and with disease related premature death. In addition, FGFR4 low expression was related with lymph node positivity and premature relapse of disease, as well as disease related death.

**Conclusion:**

Our results propose FGFR4 profile, measured by the Gly388Arg genotype and expression, as a novel marker of prognosis in squamous cell carcinoma of the mouth and oropharynx.

## Introduction

The fibroblast growth factor receptor (FGFRs) family comprises structurally related tyrosine kinase receptors (FGFR1-4) involved in signaling via interactions with fibroblast growth factors (FGFs), playing an important role in a wide range of biological processes, including differentiation, proliferation, cell motility and angiogenesis [1], [2]. Most FGFs have mitogenic activity in a variety of systems, including cell growth, differentiation and migration [1]. The proliferative capacity of FGFs is a function of FGFRs, to which they bind and through which they signal.

Deregulation in FGF/FGFR signaling has been implicated in human malignant diseases [3]–[6]. Functional studies demonstrated that FGFR4 interferes in signaling events leading to normal cell adhesiveness and corresponding invasive properties of pituitary tumors [7]. Although the molecular basis of this function is still a matter of intense research, FGFR4 seems to play a role in a broader range of human cancers [7]–[9].

A single nucleotide polymorphism (SNP) in exon 9 results in an amino acid change (substitution of a glycine residue for an arginine - Gly388Arg) within FGFR4 transmembrane domain and a positive correlation with prognostic parameters in several human cancers, including breast, colon, lung, prostate and head and neck cancers [7], [9]–[14]. Nevertheless, the association between the Gly388Arg genotype and cancer prognosis is not yet clear [15]–[18], especially in head and neck squamous cell carcinomas (HNSCC).

HNSCC ranks among the top ten most common cancers worldwide, with a large incidence variation according to sex and geographical location [19]. No biomarkers are currently available for HNSCC patients; prognosis depends largely on the stage at presentation, with the most important prognostic factor being the presence of neck node metastases [20].

To our knowledge, there is a lack of studies suggesting the prognostic significance of FGFR4 SNP genotype in HNSCC [9], [12]. Streit *et al* [9] evaluated 104 paraffin-embedded tumors and concluded that high expression of FGFR4 together with the Arg388 allele is associated with poor clinical outcome. In comparison, da Costa Andrade *et al* [12] presented results claiming an association between the FGFR4 Arg388 allele and shortened survival in 75 HNSCC patients. Given the small number of patients with tumors of different primary sites evaluated in these studies and the controversial involvement of FGFR family in human cancers, we decided to further investigate the impact the Gly388Arg polymorphism in HNSCC.

The present study evaluated the prognostic significance of FGFR4 expression and the Gly388Arg genotype in oral and oropharynx carcinomas in regard to tumor onset and prognosis. Possible correlations with clinicopathological and prognosis parameters were also analyzed.

## Materials and Methods

### Ethics

This study was approved by the Committee of Ethics in Research of the Heliopolis Hospital on 07/12/2005 (CEP # 402) and an informed consent was obtained from all patients enrolled.

### Samples

Samples were collected by the Head and Neck Genome Project (GENCAPO), a collaborative consortium created in 2002 with more than 50 researchers from 9 institutions in São Paulo State, Brazil, whose aim is to develop clinical, genetic and epidemiological analysis of HNSCC. In this study, 122 DNA and 75 tumoral tissue samples were obtained and used for polymorphism Gly388Arg genotyping and immunohistochemical analysis of the FGFR4 gene, respectively, within a total of 125 patients with oral and oropharyngeal squamous cell carcinomas, surgically treated at the Head and Neck Surgery Department of Heliópolis Hospital and Arnaldo Vieira de Carvalho Câncer Hospital, São Paulo, Brazil, during the period of January/2002 to December/2007. The clinical follow-up was at least 48 months after surgery. Previous surgical treatment, distant metastasis, no removal of cervical lymph nodes and positive surgical margins were exclusion criteria. Histopathological slides were reviewed by a senior pathologists to confirm the diagnosis and select appropriate areas for Immunohistochemical analysis. Tumors were classified according to the TNM system [21]. Clinical, epidemiological and pathological characteristics of tumors are described in Table 1.

**Table 1:** Epidemiological, clinical and pathological tumor features and their association with Gly388Arg polymorphism and FGFR4 expression.

Epidemiological, clinicaland pathological features	FGFR4									
	Genotype Gly388Arg					Expression level				
	Total		Gly/Gly	Gly/Arg+Arg/Arg	*P*value	Total		Low	High	*P*value
	No.	(%)				No.	(%)			
*Gender*										
Male	106	(86.9)	**–**	**–**	**–**	62	(82.7)	**–**	**–**	**–**
Female	16	(13.1)	**–**	**–**	**–**	13	(17.3)	**–**	**–**	**–**
*Age, yr*										
median 54, df ±10.2										
*Smoker*	98	(80.3)	**–**	**–**	**–**	54	(72.0)	**–**	**–**	**–**
*Alcoholic*	74	(60.7)	**–**	**–**	**–**	42	(56.0)	**–**	**–**	**–**
*Treatment*										
Only operated	43	(35.2)	**–**	**–**	**–**	34	(45.3)	**–**	**–**	**–**
Operated+irradiated	79	(64.8)	**–**	**–**	**–**	41	(54.7)	**–**	**–**	**–**
*Tumor sities*										
Oral cavity	87	(71.3)	**–**	**–**	**–**	60	(80.0)	**–**	**–**	**–**
Oropharynx	35	(28.7)	**–**	**–**	**–**	15	(20.0)	**–**	**–**	**–**
*Tumor size (T)*										
T1+T2	48	(39.3)	26	22	0.993	29	(38.7)	18	11	0.051
T3	31	(25.4)	17	14		19	(25.3)	9	10	
T4	43	(35.3)	23	20		27	(36.0)	22	5	
*Lymph nodes*										
Absent	59	(48.4)	35	24	0.262	31	(41.3)	16	15	0.036
Present	63	(51.6)	31	32		44	(58.7)	33	11	
*Differentiation*										
Well	47	(38.5)	24	23	0.700	32	(42.7)	24	8	0.062
Moderately	65	(53.3)	37	28		35	(46.7)	18	17	
Poorly	9	(7.4)	4	5		7	(9.3)	6	1	
Not available a	1	(0.8)				1	(1.3)			
*Lymphatic embolization*										
Negative	54	(44.3)	35	19	0.022	26	(34.7)	21	5	0.034
Positive	66	(54.1)	29	37		49	(65.3)	28	21	
Not available a	2	(1.6)				0	(0.0)			
*Perineural invasion*										
Negative	63	(51.6)	31	32	0.386	39	(52.0)	24	15	0.526
Positive	56	(45.9)	32	24		35	(46.7)	24	11	
Not available a	3	(2.5)				1	(1.3)			
*Disease specific death*										
No	55	(45.1)	37	18	0.008	40	(53.3)	21	19	0.013
Yes	44	(36.0)	18	26		31	(41.4)	25	6	
Not available a	23	(18.9)				4	(5.3)			
*Disease relapse*										
No	44	(36.1)	29	15	0.110	33	(44.0)	17	16	0.037
Yes	56	(45.9)	28	28		40	(53.3)	30	10	
Not available a	22	(18.0)				2	(2.7)			
**Total**	**122**	**(100.0)**	**66**	**56**		**75**	**(100.0)**	**49**	**26**	

a Not available (not considered in the statistical calculations).

### Genotyping

Genomic DNA was extracted from peripheral blood samples of 122 patients as previously described [22]. Genotypes were determined by polymerase chain reaction restriction fragment length polymorphism (PCR-RFLP). *FGFR4* exon 9 was amplified using primers described by Bange *et al,* [8] and analyzed for Gly388Arg polimorfism (rs351855). Selected primers were 5′ - GAC CGC AGC AGC GCC CGA GGC CAG - 3′ and 5′ - AGA GGG AAG AGG GAG AGC TTC TG - 3′ (Life Technologies, Inc®, São Paulo, SP, Brazil), which produce a 168-base pair (bp) fragment. PCR conditions were: a 25-µL reaction mixture containing 200 ng of genomic DNA, 10 mM Tris-HCl (pH 8.3), 50 mM KCl, 200 µM of each deoxyribonucleoside 5′ triphosphates, 1.5 mM de MgCl2, 1 U Taq DNA polimerase (Life Technologies, Inc®, Rockville, MD, USA) and 25 pmol of each primer. PCR initiated with a melting step of 5 minutes at 94°C, followed by 35 cycles of 1 minute at 94°C, 1 minute at 58°C and 1 minute at 72°C. PCR products were digested overnight with *BstNI* following the manufacturer’s instructions (New England Biolabs®, Berverly, MA, USA). Restriction fragments were resolved on a 12% non-denaturing polyacrylamide gel. SNP Arg388 in *FGFR4* gene was characterized by two distinctive fragments of 82 and 27 bp, whereas the *FGFR4* Gly388 wild-type allele was identified by a single fragments of 109bp.

### Tissue Microarray

Tissue microarrays were made using buffered formalin-fixed paraffin-embedded tissue sections from 75 primary oral and oropharyngeal squamous cell carcinomas treated at the Head and Neck Surgery Department of Heliópolis Hospital, São Paulo, SP, were used for immunohistochemistry (IHC) analysis. Histological characterization of all samples was done by Hematoxylin and Eosin staining, followed by immunohistochemistry analysis of tissue microarrays (TMA). Two 1 mm cylinders were used to represent each sample in the TMA slide (Beecher Instruments®, Silver Spring, MD, USA).

### Immunohistochemistry

Anti-FGFR4 monoclonal antibody (Santa Cruz Biotechnology®, USA) was used in the IHC reaction, at a 1∶400 dilution [23]–[25]. Positive (lung control) and negative controls (absence of primary or secondary antibody) were used for reaction quality control. Sample scoring was performed by semi-quantitative microscopic analysis, considering the number of stained tumor cells and signal intensity. Two spots were evaluated for each sample and a mean score was calculated. Considering the percentage of FGFR4 immune-positive tumor cells, a score of 1 was given when ≤10% of cells were positive; 2 when 10–50% and 3 when ≥50% of cells were positive. Signal intensity was scored as negative (0), weak (1), moderate (2) and strong (3). Both scores were multiplied [26], [27] and the resulting score was used to categorize FGFR4 expression as negative (≤3), low (>3 and <7) and high (>7).

### Statistical Analysis

The chi square and Fisher exact tests were used for association analysis and confirmation was obtained by the Lilliefors test (significance considered when p<0.05). Multivariate logistic regression was used to obtain odds ratio (OR) and confidence intervals (CI≥95%). Survival was calculated by the number of months between surgery and death for each patient or the last appointment in case the patient was alive. In order to calculate disease-free survival, the time endpoint was the date of disease relapse. The Kaplan-Meier model was used for survival analysis, using the Wilcoxon p-value and the Cox Proportional Hazards to adjust p-values and obtain hazard ratio (HR). Statistical calculations were performed using the Epi Info® v3.4.3, 2007 and Statsoft Statistica® v7.0.61.0 softwares. Genotype correlation with certain biological variables such as age and gender were not analyzed because we could not find biological justifications for these analyses.

## Results

### FGFR4 Gly388Arg Genotype

Regarding the SNP Gly388Arg, 66 (54.1%) cases were genotyped as Gly/Gly (wild type allele), 47 (38.5%) as Gly/Arg and 26 (7.4%) as Arg/Arg. Allele and genotype frequencies were in Hardy-Weinberg equilibrium.

The Gly388Arg polymorphism did not show a significant association with tumor size (p = 0.993), positive lymph nodes (p = 0.262) and differentiation grade (p = 0.700), but was significantly associated with lymphatic embolization (p = 0.022, Table 1). Multivariate analysis showed that presence of at least one allele Arg388 is an independent marker for lymphatic embolization (OR = 3.88, CI = 1.14–13.13, Table 2).

**Table 2:** Multivariate analysis of the relationship between clinical and pathological tumor features with gene polymorphism and FGFR4 expression.

Variables	Multivariate analysis							
	Lymphatic embolization		Lymph-nodes		Disease relapse		Disease specific death	
	OR (95% CI) ^a^	*P* value ^b^	OR (95% CI) ^a^	*P* value ^b^	OR (95% CI) ^a^	*P* value ^b^	OR (95% CI) ^a^	*P* value ^b^
*FGFR4 expression*								
High	1		1		1		1	
Low	0.46 (0.13–1.68)	0.245	3.81 (1.12–12.98)	0.032	6.73 (1.63–27.85)	0.009	6.86 (1.45–32.40)	0.015
*FGFR4 genotype Gly388Arg*								
Gly/Gly	1		1		1		1	
Gly/Arg+Arg/Arg	3.88 (1.14–13.13)	0.029	1.88 (0.60–5.83)	0.276	3.57 (0.99–12.91)	0.052	6.88 (1.64–28.87)	0.008
*Tumor size (T)*								
T1+T2	1		1		1		1	
T3	2.00 (0.45–8.84)	0.358	1.38 (0.36–5.22)	0.640	3.13 (0.67–14.57)	0.147	2.67 (0.52–13.78)	0.241
T4	1.12 (0.30–4.23)	0.859	3.16 (0.86–11.59)	0.083	1.11 (0.28–4.33)	0.885	2.31 (0.57–9.39)	0.242
*Differentiation*								
Well	1		1		–	–	–	–
Moderately	2.77 (0.83–9.18)	0.094	2.98 (0.90–9.88)	0.075	–	–	–	–
Poorly	5.70 (0.47–69.08)	0.171	3.91 (0.35–43.15)	0.266	–	–	–	–
*Lymph nodes*								
Absent	–	–	–	–	1		1	
Present	–	–	–	–	7.69 (1.21–49.00)	0.031	9.44 (1.52–58.65)	0.016
*Irradiated*								
No	–	–	–	–	1		1	
Yes	–	–	–	–	0.07 (0.01–0.50)	0.008	0.39 (0.07–2.18)	0.286

a, b Values adjusted by multivariate logistic regression.

For Gly388Arg and FGFR4 expression correlation with lymphatic embolization and lymph node status, tumor size and differentiation status were considered in the multivariate analysis. For disease relapse and disease specific death, tumor size, lymph node status and radiotherapy treatment were considered.

The Gly388Arg polymorphism was significantly associated with disease specific death (p = 0.008, Table 1) and multivariate analysis showed that presence of Arg388 allele is an independent death risk factor, increasing risk 6 times when compared to absence of this allele (OR = 6.88, CI = 1.64–28.87, Table 2). Nevertheless, the Gly388Arg polymorphism was not correlated with disease relapse (p = 0.110, Table 1).

**Figure 1:**
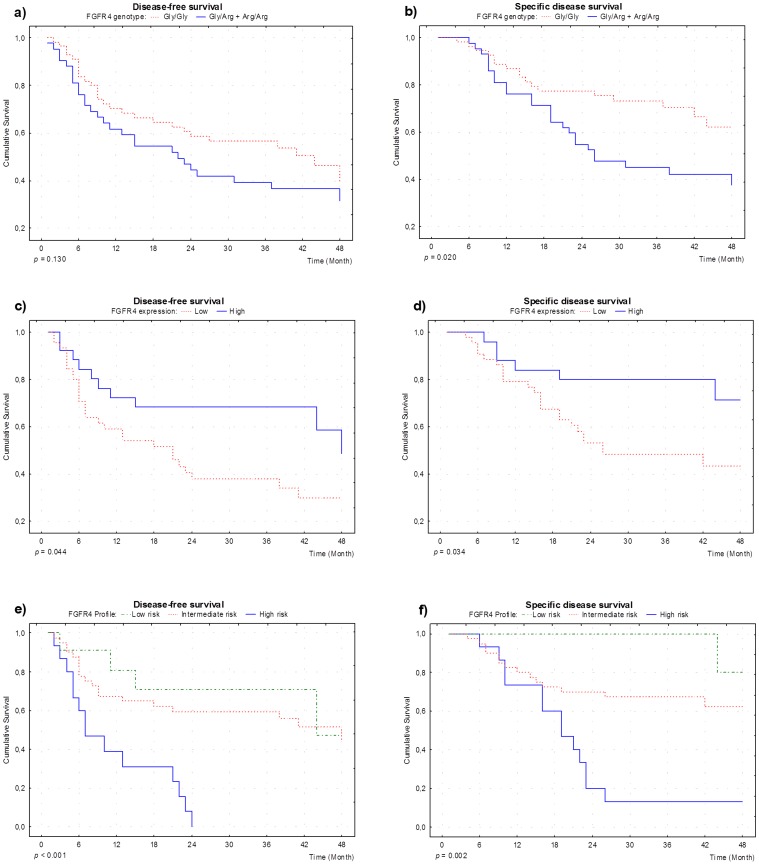
Survival plots. a. and b.: Disease-free survival and disease specific survival according to FGFR4 Gly388Arg polymorphism; c. and d.: Disease-free survival and disease specific survival according to FGFR4 expression; e. and f.: Disease-free survival and disease specific survival according to FGFR4 profile.

Although disease-free survival did not show a significant association with *FGFR4* polymorphisms (p = 0.130), presence of the Arg388 allele was associated with disease specific survival (p = 0.020). According to a 36 month after surgery follow up, approximately 25% of cases with the Gly/Gly genotype died of disease specific causes, as compared to approximately 55% of deaths in patients with the Arg388 allele (Figure 1b). Multivariate analysis revealed that the presence of Arg388 allele is an independent marker of disease specific death, with a 3 fold increased risk when compared with absence of this allele (HR = 3.26, CI = 1.40–7.58, Table 3).

**Table 3:** Multivariate analysis of disease specific survival.

Variables	Cox proportional			
	Disease-free survival		Disease-specific survival	
	HR (95% CI) ^a^	*P* value ^b^	HR (95% CI) ^a^	*P* value ^b^
*FGFR4 expression*				
High	1		1	
Low	3.26 (1.44–7.37)	0.005	3.26 (1.21–8.74)	0.019
*FGFR4 genotype Gly388Arg*				
Gly/Gly	1		1	
Gly/Arg+Arg/Arg	1.77 (0.85–3.67)	0.124	3.26 (1.40–7.58)	0.006
*Tumor size (T)*				
T1+T2	1		1	
T3	3.53 (1.46–8.52)	0.005	3.35 (1.13–9.92)	0.029
T4	1.99 (0.85–4.69)	0.115	1.65 (0.64–4.26)	0.304
*Lymph nodes*				
Absent	1		1	
Present	2.62 (1.05–6.53)	0.039	4.80 (1.56–14.73)	0.006
*Irradiated*				
No	1		1	
Yes	0.22 (0.09–6.53)	0.002	0.48 (0.18–1.27)	0.139

a, b Values adjusted by Cox proportional hazards.

Tumor size, lymp node status and radiotherapy treatment were considered in the multivariate analysis.

### FGFR4 Expression

**Figure 2:**
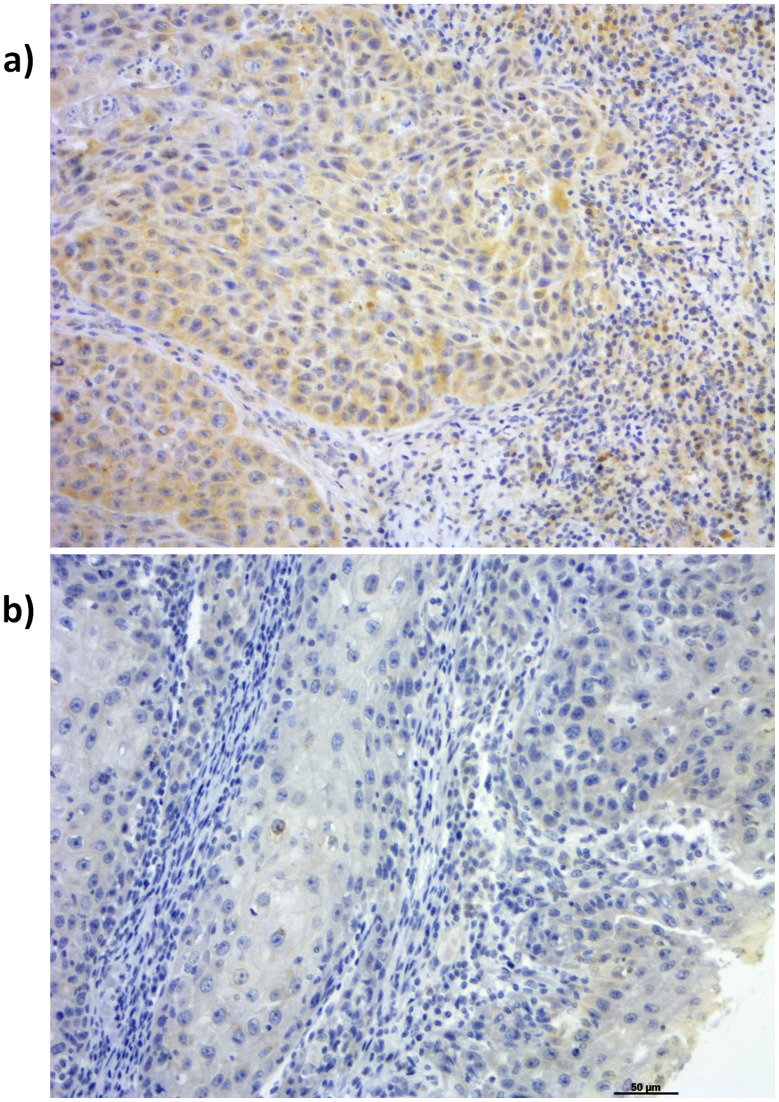
Immunohistochemical analysis of tumors. (a) strong FGFR4 expression; (b) weak FGFR4 expression. Magnification was 400×.

FGFR4 expression was detected in 75 tumors, being classified as high in 26 (34.7%) samples and low in 49 (65.3%) (Figure 2a and 2b, respectively. No samples were negative for FGFR4 expression. FGFR4 expression did not show a significant association with tumor characteristics such as size (p = 0.051) and differentiation grade (p = 0.062), but was significantly associated with positive lymph nodes (p = 0.036, Table 1). Multivariate analysis showed that low FGFR4 expression is an independent marker for lymph node positivity (OR = 3.81, CI = 1.12–12.98, Table 2). FGFR4 expression did significantly correlate with disease relapse (p = 0.037) and disease specific death (p = 0.013, Table 1). Multivariate analysis showed that FGFR4 low expression is an independent marker of disease relapse and disease specific death, representing an increased risk of over 6 times for both, in relation to high expression (respectively, OR = 6.73, CI = 1.63–27.85 and OR = 6.86, CI = 1.45–32.40, Table 2).

Disease-free and disease specific survival were significantly correlated with FGFR4 expression (p = 0.044 and p = 0.034, respectively). According to a 24 month after surgery follow up, approximately 60% of cases with low expression died of disease specific causes, as compared to approximately 30% of deaths in patients with high expression of FGFR4 (Figure 1c). Additionally, according to a 36 month after surgery follow up, approximately 50% of cases with low expression presented disease relapse, as compared to approximately 20% of recurrence in patients with high expression of FGFR4 (Figure 1d). Multivariate analysis revealed that a low expression of FGFR4 is an independent marker for a faster disease relapse and disease specific death, with a 3 fold increased risk when compared to high expression (respectively, HR = 3.26, CI = 1.44–7.37 and HR = 3.26, CI = 1.21–8.74, Table 3).

### FGFR4 Risk Profiles

In an attempt to combine genotype and expression results, we categorized the FGFR4 profile in three classes: low risk (high expression and absence of Arg388 allele); intermediate risk (high expression and presence of Arg388 allele or low expression and absence of Arg388 allele) and high risk (low expression and presence of Arg388 allele). Frequencies of each FGFR4 profile were 11 (15.5%), 43 (60.6%) and 17 (23.9%), respectively for low, intermediate and high risk.

Disease-free and disease specific survival were significantly correlated with FGFR4 profiles (p = 0.002 and p<0.001, respectively). According to a 24 month after surgery follow up, all cases classified as high risk had relapsed and approximately 80% died of disease specific causes, as compared to approximately 30% of recurrence and no deaths of patients classified as low risk (Figure 1e and 1f). Multivariate analysis revealed that the high risk category is an independent marker for a faster disease relapse and disease specific death, with a 4.5 and 13 fold increased risk, respectively, when compared to the low risk profile (HR = 4.50, CI = 1.37–14.82 and HR = 12.90, CI = 1.54–107.69).

## Discussion and Conclusions

FGFR4 belongs to the family of fibroblast growth factor receptors (FGFR1-4), transmembrane proteins with tyrosine kinase activity. Multiple signal transduction cascades are initiated after binding of FGF ligand to the extracellular domain of the receptor, ultimately resulting in gene expression changes [1], [2]. FGFRs have been shown to play important roles in several processes of embryonic development and tissue homeostasis. Their abnormal expression or mutation can cause diverse pathologies, ranging from morphogenetic disorders to cancer [28]. This is a group of proteins of considerable interest in cancer biology, because they regulate essential processes, including mitogenic and angiogenic activity, having important roles in cell differentiation, development, proliferative signaling and motility [2], [29], [30].

Several studies have examined the role of FGFR4 in carcinogenesis, providing evidences for the complexity of FGF/FGFR signaling pathways in different tumor types [7], [31]–[33].

Although the presence of *FGFR4* Arg388 allele has been shown to indicate a poor prognosis in several tumors [8], [10], [11], [34], the mechanism by which it affects cancer progression remains unclear. This might be related to signaling cascades that control cell-matrix adhesion and angiogenesis [35].

Although some mechanisms have been described in the literature, the influence of Gly388Arg polymorphism in tumor aggressiveness may differ in specific tumors.

Our study revealed that low FGFR4 expression in the presence of Arg388 allele is associated with worse survival in patients with oral and oropharyngeal squamous cell carcinoma.

Seitzer, *et al,* [36] verified, using an animal model, that low protein expression, even in the presence of FGFR4 Arg388 polymorphism, is related to increased pathway activity. This may be explained by the activation of alternative proteins in the signaling cascade or other cascades.

Recently, it has been reported that the presence of polymorphism Gly388Arg is associated with increased cancer risk and progression of pituitary tumors through recruitment of STAT3 signaling cascade. Activation of this cascade can result in deregulation of cell proliferation and apoptosis, leading to tumor progression [37]. Signaling hiperactivation by specific mutations depends on their resistance to negative feedback loops [38]. In addition, several ubiquitylation proteins bind directly to RTKs altering receptor activation [39]. RTK Ubiquitylation may promote receptor degradation creating an important negative feedback mechanism [40], [41].

FGFR4 Arg388 has not been consider an oncogene per se, but rather collaborate with oncogenes involved in cell motility and invasiveness [36].

Our findings may have important therapeutic implications, because inhibition of one intracellular pathway may lead to activation of parallel signaling pathways, thereby decreasing the effectiveness of single-agent targeted therapies [42]. In support of our hypothesis, the Arg388 allele was associated with resistance to adjuvant therapy in breast cancer [43].

Ansell *et al,* [44], were the first researchers to report that the Gly388 allele showed a significantly higher risk of developing cancer, proposing the Gly388 allele as a risk allele for head and neck cancer.

In contrast, Streit *et al,* [9] reported that in head and neck SCC, expression of Gly388 FGFR4 had no impact on disease progression. In another study, da Costa Andrade *et al,* [12] observed that the presence of at least one Arg allele was significantly correlated with reduced overall survival and an increased mortality risk of 2.2. In a recent study, Tanuma *et al,* [35] reported that *FGFR4* Arg388 allele was strongly associated with poor prognosis.

In the present study, we have shown that allele Arg388 is associated with lymphatic embolization and premature disease related death. Furthermore, low expression of FGFR4 is related to lymph node positivity and premature disease relapse and death in patients with SCC of the mouth and oropharynx.

Based on these results, we have classified patients with low FGFR4 expression/Arg388 as high risk for relapse and death. In contrast, high FGFR4 expression/Arg388-negative patients were considered at low risk. In conclusion, we propose FGFR4 profile as a novel prognostic marker in SCC of the mouth and oropharynx.
